# The art and science of study identification: a comparative analysis of two systematic reviews

**DOI:** 10.1186/s12874-016-0118-2

**Published:** 2016-02-24

**Authors:** Laura Rosen, Ruth Suhami

**Affiliations:** Deparment of Health Promotion, School of Public Health, Sackler Faculty of Medicine, Tel Aviv University, Ramat Aviv, 69978 Israel; Gitter-Smolarz Library of Life Sciences and Medicine, Tel Aviv University, Ramat Aviv, 69978 Israel

**Keywords:** Systematic reviews, Meta-analysis, Evidence-based decision making, Electronic searching, Tobacco smoke exposure, Tobacco control

## Abstract

**Background:**

Systematic reviews (SRs) form the foundation for guidelines and evidence-based policy in medicine and public health. Although similar systematic reviews may include non-identical sets of studies, and it is recognized that different sets of studies may lead to different conclusions, little work has been published on why SR study cohorts differ.

**Methods:**

We took advantage of concurrent publication of two SRs on the same topic – prevention of child exposure to tobacco smoke - to understand why study cohorts differed in the two reviews. We identified all studies included in just one review, investigated validity of specified reasons for exclusions, and, using database records, explored reasons for study non-identification. We assessed review methods and discordancy, and attempted to assess whether changes in study cohorts would have changed conclusions.

**Results:**

Sixty-one studies were included in the two reviews. Thirty-five studies were present in just one review; of these, twenty were identified and excluded by the parallel review.

Omissions were due to: review scope (9 studies, 26 %), outcomes of interest not measured (8 studies, 23 %), exclusion of reports with inadequate reporting (6 studies, 17 %), mixed or unclear reasons (3 studies, 8 %), search strategies concerning filters, tagging, and keywords (3 studies, 8 %), search strategies regarding sources (PUBMED not searched) (2 studies, 6 %); discordant interpretation of same eligibility criteria (2 studies, 6 %), and non-identification due to non-specific study topic (2 studies, 6 %). Review conclusions differed, but were likely due to differences in synthesis methods, not differences in study cohorts.

**Conclusions:**

The process of study identification for SRs is part art and part science. While some differences are due to differences in review scope, outcomes measured, or reporting practices, others are caused by search methods or discrepancies in reviewer interpretations. Different study cohorts may or may not be a cause of differing SR results. Completeness of SR study cohorts could be enhanced by 1 – independent identification of studies by at least two reviewers, as recommended by recent guidelines, 2 - searching PUBMED with free-text keywords in addition to MEDLINE to identify recent studies, and 3 - Using validated search filters.

**Electronic supplementary material:**

The online version of this article (doi:10.1186/s12874-016-0118-2) contains supplementary material, which is available to authorized users.

## Background

Systematic reviews (SRs) of the professional literature, sometimes termed the “platinum” standard of evidence [1], form the foundation for clinical guidelines and evidence-based health policy, thus shaping the policy agenda in medicine and public health [2, 3]. Contrary to narrative reviews, which may be biased due to non-systematic procedures for identifying original studies, SRs enjoy high credibility. Despite their popularity in recent years, enormous importance, and clear advantages over traditional reviews, it is not easy to validate the results of a given SR: there is simply no “gold standard” with which to compare the results. The scientific community has approached this issue by 1 – creating reporting standards [4–6]; 2 – developing tools to validate SR quality [7] and risk of bias [8]; and 3 – applying those standards and tools to published SRs [4, 9–16]. A fourth approach, made possible by the proliferation of published SRs on similar topics, is based on empirical comparisons of SR methods, results, and conclusions [17–25].

Performing a systematic review is a complex task, and searching for studies is one of the most difficult aspects. An early version of the Cochrane Handbook [Cochrane Handbook 2006, Section 5.3, p.76] [26], stated: “Identifying all relevant studies … is… largely what distinguishes a systematic review from a traditional narrative review.” Experience in conducting SRs over the years has shown that identifying all relevant studies was too ambitious; consequently, in recent versions of the handbook, that statement has been modified to read: “Systematic reviews of interventions require a thorough, objective and reproducible search of a range of sources to identify as many relevant studies as possible (within resource limits).” [27] (Section 6.1.1.2).

Indeed, identifying the entire set of relevant published studies is challenging at best, and may not be possible. In comparing SRs in 1996, Cook [19] found “incomplete identification of relevant studies”. In 17 review sets addressing the same topics, Linde found that the set of included primary studies varied by more than 50 % in 10 review sets [21]. Campbell reported differences in search strategies [18]. Rosen et al. comparing reviews performed by the US Community Guide and Cochrane on tobacco control, found that the US Community Guide did not locate (for reasons other than publication date), on average, 66.1 % of original studies included in the comparative Cochrane reviews, whereas Cochrane did not locate (for reasons other than publication date), on average, 43.5 % of Guide studies [22]. Goodyear [28] compared 2 reviews on a similar topic, and found “imperfectly overlapping studies.” Ford [25], in his study of 8 meta-analyses on similar topics, found that 6 of the 8 meta-analyses missed relevant studies, and that 5 of 8 meta-analysis included studies which were ineligible according to the review’s stated inclusion criteria. We are unaware of any previous studies comparing similar systematic reviews which focused on detailed search procedures as well as selection procedures.

On March 24th, 2014, a systematic review and meta-analysis of interventions aimed at protecting children from tobacco smoke exposure was published [29]. One author of this paper (LJR) was the lead author on that paper, and the other author (RS) had performed the literature search. The following week, on March 31, 2014, the Cochrane Collaboration published its third issue of the year, which included a review on the same topic conducted by Baxi et al. [30] While many of the studies were common to both reviews, some studies appeared in just one review. We attempted to discover, for topics which were common to both reviews, which studies were present in only one review, and find an explanation for the omission in the parallel review. Our primary goal was to identify flaws in the process of article identification in this set of two reviews, and use that information to improve future study identification processes. We also considered whether identifying or including omitted studies would have changed the results or conclusions of the reviews.

## Methods

In order to compare the sets of studies included in two reviews published almost simultaneously on the topic of child protection from tobacco smoke exposure (Rosen 2014 [29] and Baxi 2014 [30]), we did the following: 1 - Compared inclusion and exclusion criteria in the two reviews; 2 - Compared the search processes in the two reviews; 3 - Identified which studies were included in just one review; and 4 – Attempted to discover reasons for omission from one review. For studies explicitly excluded by one review, we explored why those studies were included in just one review. For studies which were not identified by one review, we examined possible reasons for non-identification. This included carefully examining records from databases to check dates of entry into the database, and keywords associated with the record.

Information on study identification, inclusion, and exclusion were obtained from the published reviews, and from correspondence with the lead author of the Cochrane review. Baxi referred to single or multiple citations, while Rosen referred to a single citation for each study. In our comparison, we used a single citation if there was one. We address issues stemming from multiple citations as necessary.

We compared the following aspects of the two reviews: methods of data synthesis; quality of reviews (AMSTAR [7]); key results and conclusions; discordancy [16]; and possible resultant policy recommendations (GRADE) [31]. We also and considered whether identification and/or inclusion of omitted studies would have changed conclusions.

## Results

### Inclusion and exclusion criteria

The differences and similarities between the inclusion criteria are presented in Table 1.

**Table 1:** Eligibility Criteria for inclusion of original studies in systematic reviews by Baxi and Rosen

Criteria	Baxi	Rosen
Title	Family and carer control programs for reducing children’s exposure to environmental tobacco smoke	Meta-analysis of parental protection of children from tobacco smoke exposure
Objectives, Abstract	“To determine the effectiveness of interventions aiming to reduce exposure of children to ETS.” (p.1)	“to quantify effects of interventions aimed at decreasing child TSE.” (p.698)
Objectives, Text	“To evaluate the effectiveness of programmes for both the prevention and cessation of smoking by those who interact with children, and teachers, and the effect on health outcomes in infants, toddlers and young children; 2) To examine and detail the indicators of intervention processes and to identify outcomes of importance to those involved in the care of children and young people.” (Objectives, p.3-4) Under Types of Interventions, she addressed aims again, with the statement: “We included studies where the primary aim was to reduce children’s exposure to ETS (thereby preventing adverse health outcomes), but where secondary outcomes included reduction or cessation of familial/parental/carer smoking, or changes in infant and child health measures. We also included studies where the primary outcome was reduction or cessation of familial/parental/carer smoking resulting in reduced exposure for children.” (p.4).	“original studies evaluating interventions aimed at protecting children from TSE.” (p.699)
Participants	Parents, caretakers, or educators of children 0-12 years.	Parents of children 0-6 years.
Interventions	No restrictions on type of intervention	No restrictions on type of intervention
Comparisons	Differences between intervention and control groups at end of study, or compared changes in outcomes from beginning to end of study, between intervention and control groups.	Differences between intervention and control groups at end of study, or compared changes in outcomes from beginning to end of study, between intervention and control groups.
Outcomes	Child exposure to ETS via biomarkers and/or parental reports, child illness, child use of health services, parental smoking behavior (including home smoking bans, cessation, change, and initiation), and cost and cost-effectiveness.	Parentally-reported exposure or protection (PREP); parentally-reported number of cigarettes smoked around child (means, standard deviations, n’s required), and biomarkers of child exposure (means, standard deviations, n’s required).
Study design	Controlled trials, with or without randomization.	Controlled trials, with or without randomization.
Time	No restrictions	Minimum 1-month follow-up

Both study titles focused on child tobacco smoke exposure (TSE) reduction. The definition of objectives as defined in the abstracts of the two reviews was essentially identical. In the text, Baxi specified objectives and outcomes which were broader than Rosen’s: she included parental/caretaker cessation and child health measures in addition to child TSE reduction. The acceptable interventions, study designs, and comparisons were identical in both reviews. Baxi’s acceptable population was broader: Rosen included parents of children aged 0-6, while Baxi included parents, caretakers, and educators of children 0–12. Rosen, but not Baxi, restricted eligibility to studies with follow-up of at least 1 month.

### Search processes

Baxi’s search strategy can be found at: http://onlinelibrary.wiley.com/doi/10.1002/14651858.CD001746.pub3/full#CD001746-sec1-0011. Rosen’s search strategy can be found in Additional file 1. The search process used by each study is summarized in Table 2. Baxi’s review was the third update of previous reviews on this topic. The updated search, most recently conducted in Sept. 2013, was conducted by a librarian and a Cochrane trial search coordinator. Rosen’s review was the second in a series of meta-analyses; the first of which focused on parental cessation [32, 33]. Rosen’s electronic search was conducted by a librarian (RS), most recently in early October, 2013.

**Table 2:** Comparison of search elements used in two systematic reviews on preventing tobacco smoke exposure in children

Search element	Baxi	Rosen
Search done by a professional librarian	Yes	Yes
Use of results of previous searches	Yes	Yes
Checking reference lists of included studies	Yes	For some studies
Enquiries for other relevant studies	Yes	No
Bibliographic Databases Searched	MEDLINE, EMBASE, CINHAL, PSYCINFO, ERIC, CENTRAL, Cochrane Specialised Group Register	PUBMED, MEDLINE, EMBASE, WoS, PSYCINFO
Use of subject headings in search strategy	Yes	Yes
Use of free text to locate relevant non-indexed reports	No	Yes
Type of filter for study design	Broad	Specific
Use of grey literature	Yes	No

Both Baxi and Rosen reported their overall search strategies, and both gave reasons for excluded studies. Rosen presented the flow chart recommended by PRISMA regarding number of records retrieved, scanned, and number of full-text articles read.

Both Baxi and Rosen searched Ovid MEDLINE, but only Rosen searched also Ovid MEDLINE In-Process & Other Non-Indexed Citations. Both used subject headings for their main subject, Rosen also used free text terms.

Both searched EMBASE, and PsychInfo. Baxi, but not Rosen, systematically reviewed reference lists of all included studies, searched the grey literature, and searched CINAHL, ERIC, CENTRAL and Cochrane Specialised Group Register. Rosen, but not Baxi, searched WoS and added Pubmed to identify articles not included in any segment of Ovid MEDLINE. Baxi used Cochrane’s highly sensitive filter for study design, while Rosen used a non-validated specific filter. Both authors used published and unpublished information obtained from the authors.

### Study identification and exclusion

#### All studies identified

Sixty-one studies were included in the two reviews. Baxi included 57 studies, and Rosen included 30 studies. Twenty-six studies were included in both reviews (Cochrane Identifier: Abdullah 2005, Baheiraei 2011, Butz 2011, Chan 2006a, Chellini 2013, Conway 2004, Eriksen 1996, Fossum 2004, Greenberg 1994, Groner 2000, Halterman 2011, Hovell 2000, Hovell 2002, Hovell 2009, Irvine 1999, Krieger 2005, McIntosh 1994, Prokhorov 2013, Severson 1997, Stotts 2012 2013, Tyc 2013, Wakefield 2002, Wilson 2001, Wilson 2011, Yilmaz 2006, Zakarian 2004). The Cochrane review provided multiple citations for many of the studies. One study which was included in both reviews was based on information from different reports [34, 35].

**Fig. 1:**
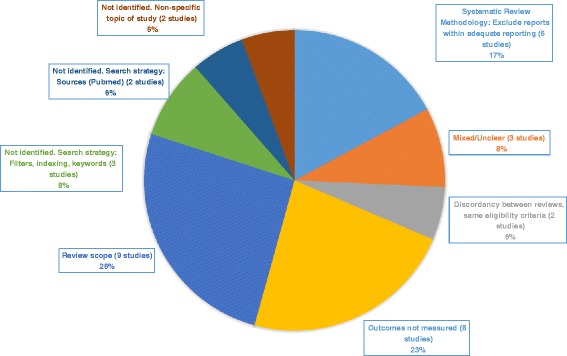
Reasons for omission of studies from one of two revews

The thirty-five studies which were included in just one review are listed in Table 3 [36–70]. Figure 1 summarizes the status and reasons for omission of all studies.

**Table 3:** List of all studies included in one of the two reviews

Study	Status Baxi	Status Rosen	Reason for exclusion / Failure to identify study	Reason for omission
Armstrong 2000	Included	Not identified	Non-specific study topic	Not identified: Non-specific study topic
Borelli 2010	Included	Identified & Excluded (FT)	No true control group	Discordant interpretation of same eligibility criteria
Chan 2005	Included	Identified & Excluded (FT)	Outcomes	Outcomes not measured
Chilmonczyk 1992	Included	Identified & Excluded (FT)	Data unavailable	Systematic review methodology: Exclude reports with inadequate reporting
Culp 2007	Included	Identified & Excluded (ABS)	Population (pregnant)	Review scope
Curry 2003	Included	Identified & Excluded (FT)	Outcomes	Outcomes not measured
Davis 1992	Included	Not identified	Review scope	Review scope
Ekerbicer 2007	Included	Identified & Excluded (TI)	Population (age)	Review scope
Elder 1996	Included	Not identified	Search strategy: Search limits (age filter)	Review scope
Emmons 2001	Included	Identified & Excluded (FT)	Outcomes	Outcomes not measured
French 2007	Included	Not identified	Review scope	Review scope
Hannover 2009	Included	Not identified	Review scope	Review scope
Herbert 2011	Included	Identified & Excluded (FT)	Data unavailable	Systematic review methodology: Exclude reports with inadequate reporting
Huang 2013	Not identified	Included	Search strategy: Source (PUBMED not searched)	Not identified: Search strategy: Source (PUBMED not searched)
Hughes 1991	Included	Identified & Excluded (FT)	Outcomes	Outcomes not measured
Kallio 2006	Included	Identified & Excluded (FT)	Data unavailable	Systematic review methodology: Exclude reports with inadequate reporting
Kimata 2004	Included	Identified & Excluded (FT)	Data unavailable	Systematic review methodology: Exclude reports with inadequate reporting
Lanphear 2011	Identified & Excluded (ABS)	Included	Goals	Discordant interpretation of same eligibility criteria
Nuesslein 2006	Included	Identified & Excluded (ABS)	Outcomes	Outcomes not measured
Patel 2012	Included	Not identified	Search strategy - Excluded by filter due to incorrect tagging	Not identified: Search strategy: Filters, tagging, keywords
Philips 2012	Included	Not identified	Review scope	Review scope
Pulley 2002	Included	Not identified	Search strategy: inadequate reporting by authors	Not identified: Search strategy: Filters, tagging, keywords
Ralston 2008	Included	Identified & Excluded (FT)	Outcomes	Outcomes not measured
Ralston 2013	Included	Identified & Excluded (ABS)	Outcomes	Outcomes not measured
Ratner 2001	Included	Identified and Excluded (FT)	No true control group	Mixed/Unclear
Schonberger 2005	Included	Identified & Excluded (FT)	Data unavailable	Systematic review methodology: Exclude reports with inadequate reporting
Streja 2012	Not identified	Included	Search strategy: Source (PUBMED not searched)	Not identified: Search strategy: Source (PUBMED not searched)
Teach 2006	Not included or excluded	Included	Unclear status	Mixed/Unclear
Van't Hof 2000	Included	Not identified	Review scope	Review scope
Vineis 1993	Included	Not identified	Search strategy - Excluded by filter due to incorrect tagging	Not identified: Search strategy: Filters, tagging, keywords
Wahlgren 1997	Included	Identified & Excluded (FT)	Follow-up trial	Mixed/Unclear
Wiggins 2005	Included	Not identified	Non-specific study topic	Not identified: Non-specific study topic
Winickoff 2010	Included	Identified & Excluded (FT)	Outcomes	Outcomes not measured
Woodward 1987	Included	Identified & Excluded (FT)	Data unavailable	Systematic review methodology: Exclude reports with inadequate reporting
Zhang 1993	Included	Not identified	Search strategy: Search limits (age filter)	Review scope

Of the 35 studies, twenty were identified by the parallel review and excluded [37–41, 43, 45, 48, 50–54, 58–61, 66, 68, 69]. All but one of these [53] were found and excluded by Rosen.

#### Studies identified and excluded

Baxi identified and excluded one study (Lanphear [53]) at the abstract stage, on the basis of goals. The stated reason for the exclusion was that reducing child environmental tobacco smoke exposure was not defined as a primary objective (Personal communication, July 31, 2014). However, in the Introduction, Lanphear included secondhand smoke exposure reduction as an explicit goal. The reason for omission was categorized as “Discordant interpretation of same eligibility criteria.” The remaining 19 studies were excluded by Rosen. One study was excluded at the title stage (Ekerbicer [43]), due to age of children. Three studies were excluded at the abstract stage; one due to age (Culp [40]), one due to outcomes and goals (maternal tobacco consumption) (Nuesslein [54]), and one due to outcomes (Ralston 2013 [58]). Of the 15 studies which were excluded by Rosen at the stage of full-text reading, 6 were excluded because there were no relevant outcomes. (Chan [38], Curry [41], Emmons [45], Hughes [50], Ralston 2008 [58], Winickoff [68]) Six studies were excluded because data necessary for meta-analysis review was missing [39, 48, 51, 52, 61, 69]. All but one of these [61] were missing means, and/or standard deviations, and/or sample sizes.

The exclusion of one study after reading the full-text of the reports was based on discordant interpretation for the same eligibility criteria (study design) [37]. This occurred even though both reviewers had identical inclusion criteria for study design. The Borrelli study design involved randomization to 2 active intervention groups. Rosen excluded the study because it was unclear which group to include as the “intervention” and which to include as the “control”.

Rosen excluded one paper [66] because it was a follow-up study of a paper by Hovell 1994 [71] which she was planning to include. After the initial exclusion decision on the Wahlgren paper was made, the Hovell trial was excluded because data for the meta-analysis were unavailable from the authors. Had the Wahlgren paper been reconsidered (as it should have been); it would have been rejected because the control group was exposed to intervention materials at the close of the initial intervention period, leading to the probability that the control group was contaminated by exposure to control materials. This was categorized as “mixed” reason for omission.

The Ratner paper [60] was excluded by Rosen due to lack of a control group. The paper described a small, uncontrolled observational study which was a follow-up of a randomized trial. That study was included by Baxi, who cited 5 papers relating to that trial, which focused on preventing smoking relapse post-partum. The additional reports were not found by, or relevant to, Rosen’s review. This could not be clearly classified as a discrepancy between reviewers, as it is possible that Baxi primarily utilized data from the other quoted studies. Therefore the reason for omission was categorized as “Mixed”.

#### Studies not identified

Fourteen studies were not identified by the parallel review [36, 42, 44, 46, 47, 49, 55–57, 62, 64, 65, 67, 70].

Two of these were excluded automatically by Rosen’s age filter [44, 70]. Two studies [49, 62] were not found by Baxi because of her search methodology: she did not search PUBMED, and these two publications were available in PUBMED, but not MEDLINE, at the time of search.

The following three articles, which appeared in Baxi’s review, but were not identified by Rosen’s electronic search, were not identified due to issues with filters, index terms or free-text search terms:

- The Patel study [55] was in EMBASE at the time of Rosen’s search. Though the Abstract and Methods sections stated that participants had been randomized to intervention or control groups, and the study design was therefore a randomized controlled trial (RCT), there was no mention of RCT in the Abstract or title. Rather, the authors used the term “prospective follow-up Pilot study” in the Methods Section of the article. The article was not indexed as an RCT.
- Rosen’s search filter relied on correct indexing (“randomized controlled trial” or “controlled clinical trial” as Emtree terms). Baxi found Patel in the Cochrane register, where it had been identified through a periodic search of EMBASE.
- The Vineis study [65] was missed by Rosen’s MEDLINE search. Vinies called his study a “population-based trial” in the Abstract, and a “non-randomized experimental design” in the Methods section. Participants were assigned to intervention and control groups in a non-random manner. This was therefore a controlled trial, but was not termed as such by the authors.
- The Pulley study [57], which was in PUBMED but not indexed in MEDLINE at the time of the search, was missed because neither the title nor the abstract mentioned that it was an RCT. Rather, it was described a “longitudinal, quasi-experimental design” in both the Abstract and the Methods section. Confirmation that this was an RCT is in the Methods section, in the statement: “Mother-infant pairs were randomly assigned to the either the control or intervention group (p.31). In addition, the status of this record in PUBMED is “Pubmed-not-Medline” which means that this record is not indexed.

Five studies which were included by Baxi but not identified by Rosen addressed topics not included in Rosen’s review (prevention of postpartum relapse to smoking [46, 47, 56, 64]; cessation among young mothers [42]). Two additional studies which were included by Baxi but not identified by Rosen [36, 67], addressed very general health outcomes, without a stated objective regarding protection of children from tobacco smoke. One of those papers [36] was identified during a search for papers for a review on another topic. Another was identified in previous versions of the Cochrane review, (Personal correspondence, Ruchi Baxi, 31 July 2014) but not through an electronic search for this review.

#### Duplicate / unclear study identification

One study which was included by Rosen but not by Baxi [63] may have been identified and excluded prior to reading the full-text article, but it is unclear (Personal correspondence, Baxi, July 31, 2014).

Another study was included in both reviews, but based on two different reports [34, 35]. Baxi included a conference report [34] which Rosen did not locate, as she did not search grey literature sources. Rosen included a published paper [35] which was not identified by Baxi because she did not search PUBMED, and at the time of search the paper was available on PUBMED but not on MEDLINE.

### Summary of reasons for omissions

We found that omissions were attributable to: review scope (9 studies, 26 %) [40, 42–44, 46, 47, 56, 64, 70], outcomes of interest not measured (8 studies, 23 %) [38, 41, 45, 50, 54, 58, 59, 68], exclusion of reports with inadequate reporting (6 studies, 17 %) [39, 48, 51, 52, 61, 69], mixed or unclear reasons (3 studies, 8 %) [60, 63, 66], search strategies concerning filters, tagging, and keywords (3 studies, 8 %) [55, 57, 65], search strategies regarding sources: PUBMED not searched (2 studies, 6 %) [49, 62]; discordant interpretation of same eligibility criteria (2 studies, 6 %) [37, 53]; and non-identification due to non-specific study topic. (2 studies, 6 %) [36, 67].

### Comparison of the two reviews

- *Data synthesis*

Data were synthesized differently in the two reviews. Baxi used a narrative approach to synthesizing the reviews, noting that this was because of heterogeneity of methodologies and outcome measures; she used the “head-counting” [72] approach to determining how many studies showed statistically significant results. Rosen took a meta-analytic approach. She used the random effects model due to heterogeneity between studies, and standardization to overcome the problem of heterogeneity between outcome measures. Using a narrative approach, as was done by Baxi, and using random effects models, as was done by Rosen, are both considered reasonable solutions to the problem of heterogeneity [73].

b. *Assessment of quality using AMSTAR*

The AMSTAR [7, 73] checklist was used to assess quality of the two SRs. Baxi’s review received a perfect score (11/11). Rosen received a score of 9/11. She lost one point because she did not have a published protocol and one point because she did not search the grey literature.

c. *Comparisons of key results and conclusions*

Review conclusions differed for both primary and subgroup analyses.

#### Primary analysis

Baxi: The Results Section of the Abstract reported that “In only 14 of the 57 studies was there a statistically significant intervention effect for child ETS exposure.” In the Authors’ Conclusions Section of the Plain Language Summary, Baxi interpreted this and her other statements to mean that “Although several interventions …. have been used to try to reduce children’s tobacco smoke exposure, their effectiveness has not been clearly demonstrated.”

Rosen: Rosen’s results showed that interventions demonstrated some benefit to intervention participants at follow-up for parentally-reported exposure or protection (PREP outcome) (relative risk 1.12, *p* < .0001) and number of cigarettes smoked around children by parents at follow-up (*P* = .03). There was a non-significant trend towards benefit to interventions as measured by biomarkers ((RD 20.05, CI 20.13 to 0.03, *P* = .20). The summary statement in the Conclusions section of the abstract stated: “Interventions to prevent child TSE are moderately beneficial at the individual level.”

Using Moja’s [16] approach for discordant findings, Rosen’s review could be classified as “efficacious” because there was a statistically significant benefit to the intervention groups when using the parentally-reported measures, or as “mixed” as biochemical measures did not show a statistically significant effect.

The two SRs would probably be considered discordant by Moja’s criteria.

#### Subgroup analysis

Baxi: Baxi found that “The review was unable to determine if any one intervention reduced parental smoking and child exposure more effectively than others, although seven studies were identified that reported intensive counselling or motivational interviewing provided in clinical settings was effective.” In the Conclusions Section of the text, she stated that “no intervention or setting was clearly more efficacious, and that intensive interventions for parents showed limited success.”

Rosen: In an exploratory subgroup analysis, Rosen found that “Most subgroups showed significant, albeit small, benefit to the interventions.”

Neither author had a very clear statement, as Baxi’s was ambiguous, and Rosen’s was based on exploratory analyses.

d. *Translation into policy recommendations using GRADE.* The GRADE system [31] provides guidance in how to use evidence to make recommendations. There are four possible recommendations: strong against, strong for, weak against, and weak for. Given the positive finding of Rosen on parentally-reported measures, and non-significant finding on biochemical measures, it is unclear what the classification would be. Baxi’s results might result in a “research only” recommendation.
e. *Would identification and/or inclusion of studies omitted have changed review conclusions?*

It is not possible to give a definitive answer to this question, but we tried to assess the possible impact of the unidentified studies.

- ROSEN: Of the studies not identified by Rosen, three would have been excluded on the basis of inclusion criteria (age: Elder [44], Zhang [70], minimum follow-up: Pulley [57]), five on the basis of goals (postpartum relapse: French [46], Hannover [47], Phillips [56], Van’t Hopf [64], cessation of young mothers Davis [42]), one due to broad goals with no measured outcomes of interest (Wiggens [67]), two due to poor reporting (no reports on relevant outcomes at study end for all randomized participants (Vineis [65], Patel [55]). One study (Armstrong [36]) had general goals and included relevant data; whether it would have been included would have required a judgement call. Therefore, the maximum difference which could have occurred would have been the addition of one study to one of the four endpoints examined by Rosen. The meta-analysis was rerun, with nearly identical results (RR = 1.13, p < .0001). Therefore, identification of additional studies would not have affected review results.
- BAXI: Two studies (Huang [49] and Streja [62]) included by Rosen were not identified by Baxi, an additional one (Teach [63]) may or may not have been identified, and one more (Lanphear [53]) may have been mistakenly omitted. Baxi (personal communication, July 31, 2014) thought that of these studies, only Streja [62] would have been included. It is not clear how this would have been handled by two Cochrane reviewers. The maximum difference in results would come from adding all four studies to the review. Two of the studies, Huang [49] and Teach [63], showed beneficial and statistically significant benefit to the intervention on parentally-reported child exposure or protection (PREP) at study end. Streja [62] did not show a statistically significant benefit on change in PREP. Lanphear [53] found no differences between intervention and control groups on child biomarkers at study end or on numbers of cigarettes smoked around the house.

If the Steja study was included in the review, then 14/58 instead of 14/57 studies would have been shown to be beneficial. The addition of all 4 studies- the maximum change - would have resulted in statistically-proven benefit in 16/61 (26.2 %) of studies instead of 14/57 studies (24.6 %). This would not have changed the main conclusions of the review.

## Discussion

The process of identifying studies for inclusion in a systematic review is complex, and involves both electronic and human aspects. Of the sixty-one studies included in the two reviews analyzed in this report, nearly 60 % (35/61) were present in just one review. Of these, over half (20/35), were identified and excluded by the parallel review, while over 40 % were not identified. Most omissions were due to differences in review scope (as expressed in inclusion criteria and search filters), measurement of outcomes, differing requirements for quantitative data, and search issues, including how and which sources were searched. A minority of omissions (2) resulted from discordant reviewer interpretations of identical inclusion criteria. We explore these issues below.

### Review scope

Review scope was an important factor in creating the different study cohorts in these reviews, as expressed in search strategies and inclusion criteria. It has been noted that differing inclusion criteria is one of the factors which contributes to SR discordance [15]. In our comparison, omission of studies from one review was sometimes due to differences in inclusion criteria, which were much broader in Baxi’s review than in Rosen’s review. Differences in age criteria (up to age 12 in Rosen, and up to age 18 in Baxi) caused the exclusion of two studies [40, 43] and were likely responsible for the non-identification of two additional studies [44, 70]. The scope of the review was like also responsible for the non-identification of an additional 5 studies [42, 46, 47, 56, 64]. which would have been excluded from Rosen’s review.

### Exclusions due to measurement and reporting of outcomes

The Cochrane MECIR best practice guidelines, state that neither outcomes nor poor reporting should be used as an exclusion criteria. Inclusion of these studies in the review, even if some studies won’t be used in the meta-analysis, is desirable because it allows readers to judge whether outcome reporting bias exists. The MECIR guideline recommends that “If authors do exclude studies on the basis of outcomes, care should be taken to ascertain that relevant outcomes are not available because they have not been measured rather than simply not reported.”

Missing relevant outcomes caused the exclusion of 8 studies by Rosen [38, 41, 45, 50, 54, 58, 59, 68]. Other studies not included in this comparison were excluded from both Rosen and Baxi due to outcomes. The absence of key data from six studies [39, 48, 51, 52, 61, 69] caused the exclusion of those studies from Rosen’s review, which employed a meta-analytic approach, but not from Baxi’s review, which used a narrative approach to synthesizing the data. Rosen differentiated between studies which didn’t collect outcomes (“no relevant outcomes reported”) and studies which didn’t report on relevant outcomes (“missing data”).

### Electronic search issue 1: Search dates, MEDLINE, and PUBMED

Though MEDLINE and PUBMED are sometimes thought to be identical, they are not: According to the U.S. National Library of Medicine, as of May 2014, MEDLINE held over 21 million records, while PUBMED had over 23 million references, including all MEDLINE records and additional records [74]. Some of the 2 million additional references refer to very recent publications which are not yet available in MEDLINE, while others refer to articles in journals not covered by MEDLINE. The reason why articles may appear in PUBMED earlier than MEDLINE is a function of the process of article entry into the two databases. When an article is first published electronically, publishers can upload it immediately to PUBMED, prior to print publication. The record receives the status of "Publisher" in PUBMED records, and at this stage it appears in PUBMED only, and does not yet appear in any segment of MEDLINE [75]. The record goes through two additional stages before it fully enters into MEDLINE: first, the record receives a status of "In-Data Review," during which time the article data are validated; then, the record receives a status of "In Process" until MESH index terms are assigned. At this stage the record can also be found by searching Ovid MEDLINE In-Process database using free-text keywords.

This indexing process accounts for the time lag between appearance of a record in PUBMED and its appearance in MEDLINE. The time lag can be considerable. Duffy [76] investigated the time lags of two studies and found out that one study included in a SR was available in MEDLINE a month after it appeared in PUBMED, while a second study that was included in an SR did not appear in MEDLINE until six months after it appeared in PUBMED.

Three recent reports were missed by Baxi [35, 49, 62], who searched MEDLINE but not PUBMED, but were found by Rosen through her PUBMED search with free-text search terms. One of these studies [34] was included in abstract form in Baxi’s review. The time period for Stotts 2013 [35] to move from "Publisher" status to "In-Process" and enter MEDLINE was five months, the time period for Huang 2013 [49] was more than 8 months. Streja [62] entered MEDLINE only a year and a half after being available in PUBMED.

### Electronic search issue 2: Indication of study design in title or abstract

In bibliographic databases such as MEDLINE, PUBMED and Embase, searches are done on the title, abstract and subject headings, not on the full-text of the article. When a record is not indexed, the ability to filter for study design is dependent upon correct reporting in the title and/or abstract of the report. One study [57], a “pubmed-not-medline” record, was missed because neither the title nor the abstract indicated that it was an RCT.

### Electronic search issue 3: Index terms, free-text terms, and search filters

Index terms are assigned to articles in MEDLINE (MESH), EMBASE (EMTREE), and other databases. They describe the subject of the study and other parameters such as study design. Study design can serve as a filter to the search when a SR is limited to a certain study design, such as RCT. In this study, two reports were missed by Rosen due to problems with index terms [55, 65].

The indexing process is a complex task in which judgment calls by indexers play an important role. Indexing problems are not uncommon. Crumley [77] indicates that "For electronic databases, the reason cited most often (67 %) for missed studies was inadequate or inappropriate indexing". Our findings suggest that inadequate indexing is due at least in part to author failure to include study design explicitly in the title or abstract. Both the Cochrane Handbook [27] and the CONSORT reporting guidelines [78] require reporting of study design in the title or abstract. Our findings, and information about missed studies in the literature, support these recommendations.

For this reason, Cochrane Handbook and other guidelines also recommend using free-text terms in addition to index terms for the search process.

In order to help researchers combine their subject search terms with appropriate study design, methodological search filters were developed and tested for their quality. Cochrane developed filters for RCT in MEDLINE, (http://handbook.cochrane.org/ Section 6.4.11) but other organizations developed their own. For example, SIGN, a Scottish guidelines body, develops in-house filters and adapts other organizations' filters according to its needs. SIGN states that its filters "may provide less sensitive searches than used by other systematic reviewers such as The Cochrane Collaboration, but enable the retrieval of medical studies that are most likely to match SIGN's methodological criteria." (http://www.sign.ac.uk/methodology/filters.html#random) BMJ Clinical Evidence presents a different filter for RCT (http://clinicalevidence.bmj.com/x/set/static/ebm/learn/665076.html). CADTH, a Canadian organization that publishes evidence-based medicine research, also develops and maintains its own search filters (https://www.cadth.ca/resources/finding-evidence/strings-attached-cadths-database-search-filters).

Over the years, numerous filters have been developed, and one study compared between as many as 38 RCT filters for MEDLINE [79]. These filters differ in the way and the time they were developed, tested and validated, and their performance is not always well reported [80].

Best choice of filter is difficult, and a clear guidance or tool to help researchers in this complex task is lacking. A recent study suggested that filter performance be presented as a forest plot, to allow visualization by reviewers of benefits [81]. Though there is no consensus on how to choose a filter, using a validated filter is recommended.

### The ubiquitous role of judgment calls

Even with clearly defined inclusion and exclusion criteria, and an appropriate search strategy, reviewers must regularly make judgment calls about whether to include or exclude a study. This occurs at all stages of the process of study identification: at the level of scanning records, at the level of reading abstracts, and at the level of reading full-text articles. It has previously been suggested that bias can be “injected” into meta-analysis at the stages of finding studies, selection of studies for inclusion, and data extraction [82].

In our comparison, two identified studies were omitted on the basis of differences in judgments between reviewers [37, 53]. We did not find reason to suspect bias. Rather, these examples illustrate the complexity of judgement calls.

The responsibility for making reasonable decisions falls on different individuals, at different points in the process. Errors might arise due to the creation of the search strategy, which may be under the control of one or more individuals; by authors of original reports, as they decide how to write the report and which data to include; by database indexers, and by reviewers.

### Possible effects of missing studies on review results and conclusions

There are several possible effects of omitting studies from systematic reviews. First, such omissions reduce usefulness, because the review is incomplete. Second, such omissions could affect conclusions, particularly if many relevant studies were missed. Even a small number of missed studies – even if the results are in the same direction as the results of the found studies - may affect conclusions if the study estimates are synthesized using a meta-analytic approach: fewer studies result in a loss of power to detect a true effect if it exists. Third, such omissions may suggest bias in the results, either intentional (See: Goodyear-Smith [28]) or unintentional. The smaller bias in SRs with more comprehensive search strategies has been previously noted [15].

### Would the review results have changed with the identification of or inclusion of omitted studies?

Our analyses showed that identification and inclusion of omitted studies would likely not have changed results of either review. The most likely reason for discrepant results is due to differences in summary methods. Differences in scope may have also played a role, though that is difficult to show, as the summary methods differed. It is well known that one of the advantages of meta-analytic synthesis over narrative synthesis is that small effects may be detected and quantified. The heterogeneity found by both reviews was dealt with in different ways, and loss of ability to detect true effects was the main downside of the narrative summary approach taken by Baxi.

### Strengths and limitations

This work presents a unique approach to exploring why cohorts of original studies in similar systematic reviews differ. Unlike the few previous comparisons which explored reasons for discrepancies in SR cohorts [19, 21, 28], this study focused on the study identification process, and used detailed, date-specific information from MEDLINE, PUBMED, and EMBASE to understand why some studies were missed. The publication of two reviews almost simultaneously on the same topic, with similar dates of searching, allowed the comparison.

Although only two reviews were included in the comparison, we were able to identify several lacunae in the SR study identification and selection process. Because our findings about omissions are general in nature, they are likely to be problematic in some other systematic searches as well. More work is necessary to assess how prevalent these issues are. Adopting the comparative approach used here will likely yield information on other stumbling blocks to identification of all relevant studies for SRs. Combining knowledge from such comparisons could serve as an engine for improved methods to identify relevant studies.

### Recommendations

Our recommendations for enhancing complete identification of relevant studies for systematic reviews can be found in Table 4 below.

**Table 4:** Recommendations for enhancing complete identification of relevant studies for systematic reviews

1. This study provides empirical support for recommendations by recent systematic review guidelines, which state that two reviewers should independently identify studies.
2. Reviewers should search pubmed with free-text terms to identify recent studies, in addition to searching medline, due to the lag-time between article publication and entry to medline.
3. When using a study design as part of the search strategy, validated search filters are preferable

## Additional file

Additional file 1:
**Rosen’s Meta-analysis MEDLINE Search Strategies.** (DOCX 14 kb)

## Abbreviations

SR: Systematic Review
TSE: Tobacco smoke exposure
RCT: Randomized controlled (or clinical) trial
