# Supplementary material for: The art and science of study identification: a comparative analysis of two systematic reviews
Source: BMC Med Res Methodol. 2016 Feb 24;16:24. doi: 10.1186/s12874-016-0118-2 (PMC4766738; doi:10.1186/s12874-016-0118-2)
Supplement: Additional file 1: — Rosen’s Meta-analysis MEDLINE Search Strategies. (DOCX 14 kb) [file 12874_2016_118_MOESM1_ESM.docx]

Appendix 1- Rosen's Meta-analysis MEDLINE Search Strategies

Database: Ovid MEDLINE(R) <1946 to September Week 4 2013>

Search Strategy:

1 Tobacco Smoke Pollution/ (10727)

2 (second-hand smok* or secondhand smok*).mp. [mp=title, abstract, original title, name of substance word, subject heading word, keyword heading word, protocol supplementary concept, rare disease supplementary concept, unique identifier] (1870)

3 passive smok*.mp. [mp=title, abstract, original title, name of substance word, subject heading word, keyword heading word, protocol supplementary concept, rare disease supplementary concept, unique identifier] (3604)

4 environmental tobacco smok*.mp. [mp=title, abstract, original title, name of substance word, subject heading word, keyword heading word, protocol supplementary concept, rare disease supplementary concept, unique identifier] (3075)

5 involuntary smok*.mp. [mp=title, abstract, original title, name of substance word, subject heading word, keyword heading word, protocol supplementary concept, rare disease supplementary concept, unique identifier] (90)

6 Tobacco smoke exposure.mp. [mp=title, abstract, original title, name of substance word, subject heading word, keyword heading word, protocol supplementary concept, rare disease supplementary concept, unique identifier] (1256)

7 exp Parents/ (72626)

8 exp "Tobacco Use Cessation"/ (21782)

9 7 and 8 (296)

10 1 or 2 or 3 or 4 or 5 or 6 (13329)

11 randomized controlled trial/ (387443)

12 controlled clinical trial/ (89701)

13 child/ or child, preschool/ or infant/ or infant, newborn/ (2018130)

14 11 or 12 (472223)

15 9 or 10 (13499)

16 13 and 14 and 15 (163)

17 case-control studies/ or cross-sectional studies/ (356731)

18 16 not 17 (150)

19 limit 18 to (meta analysis or systematic reviews) (2)

20 18 not 19 (148)

21 (protocol or observational or guideline*).ti. (72873)

22 20 not 21 (144)

***************************

Database: Ovid MEDLINE(R) In-Process & Other Non-Indexed Citations <October 01, 2013>

Search Strategy:

1 (second-hand smok* or secondhand smok*).mp. [mp=title, abstract, original title, name of substance word, subject heading word, keyword heading word, protocol supplementary concept, rare disease supplementary concept, unique identifier] (215)

2 passive smok*.mp. [mp=title, abstract, original title, name of substance word, subject heading word, keyword heading word, protocol supplementary concept, rare disease supplementary concept, unique identifier] (160)

3 environmental tobacco smok*.mp. [mp=title, abstract, original title, name of substance word, subject heading word, keyword heading word, protocol supplementary concept, rare disease supplementary concept, unique identifier] (129)

4 involuntary smok*.mp. [mp=title, abstract, original title, name of substance word, subject heading word, keyword heading word, protocol supplementary concept, rare disease supplementary concept, unique identifier] (2)

5 Tobacco smoke exposure.mp. [mp=title, abstract, original title, name of substance word, subject heading word, keyword heading word, protocol supplementary concept, rare disease supplementary concept, unique identifier] (64)

6 (newborn$ or infant$ or child or children).mp. [mp=title, abstract, original title, name of substance word, subject heading word, keyword heading word, protocol supplementary concept, rare disease supplementary concept, unique identifier] (52066)

7 randomized controlled trial/ (424)

8 controlled clinical trial/ (24)

9 random$.ti,ab. (53968)

10 (clin$ adj1 trial$).ti,ab. (14725)

11 (Controlled adj2 trial$).ti,ab. (10351)

12 7 or 8 or 9 or 10 or 11 (64481)

13 1 or 2 or 3 or 4 or 5 (487)

14 6 and 12 and 13 (27)

15 (cross-section* or case-control or meta-analysis or systematic review or protocol).ti. (11998)

16 14 not 15 (21)

17 (observational or guidelines).ti. (3526)

18 16 not 17 (20)
